# Heterogenous NECTIN4 expression in urothelial high-risk non-muscle-invasive bladder cancer

**DOI:** 10.1007/s00428-022-03328-1

**Published:** 2022-04-28

**Authors:** Stefan Garczyk, Stephan Degener, Felix Bischoff, Tician Schnitzler, Anne Salz, Reinhard Golz, Alexander Buchner, Gerald B. Schulz, Ursula Schneider, Nadine T. Gaisa, Ruth Knüchel

**Affiliations:** 1 Institute of Pathology, University Hospital RWTH Aachen, Aachen, Germany; 2 Center for Integrated Oncology Aachen Bonn Cologne Duesseldorf (CIO ABCD), Aachen, Germany; 3 Department of Urology, Helios University Hospital Wuppertal, Wuppertal, Germany; 4 Institute of Pathology, Helios University Hospital Wuppertal, Wuppertal, Germany; 5 Department of Urology, Ludwig-Maximilians-University Munich, Munich, Germany

**Keywords:** NMIBC, Bladder cancer, NECTIN4, Intratumoral heterogeneity, Therapy

## Abstract

High-grade non-muscle-invasive bladder cancer (HG NMIBC) patients are at high risk (HR) of progression to muscle-invasion. Bladder-preserving therapies for this patient subgroup are limited, and additional treatments are desirable. Recently, enfortumab vedotin, targeting cancer-associated NECTIN4, has been approved for the treatment of advanced urothelial carcinoma. However, data on the expression of NECTIN4 and its therapeutic potential for HR NMIBC are scarce. Here, NECTIN4 was immunohistochemically analyzed in urothelial HG NMIBC by studying cohorts of carcinoma in situ (CIS)/T1HG (N = 182 samples), HG papillary tumors from mixed-grade lesions (mixed TaHG) (N = 87) and papillary HG tumors without a history of low-grade disease (pure TaHG/T1HG) (N = 98) from overall 225 patients. Moreover, inter-lesional NECTIN4 heterogeneity in multifocal HG NMIBC tumors was determined. A high prevalence of NECTIN4 positivity was noted across HG NMIBC subgroups (91%, N = 367 samples), with 77% of samples showing moderate/strong expression. Heterogenous NECTIN4 levels were observed between HG NMIBC subgroups: non-invasive areas of CIS/T1HG and pure TaHG/T1HG samples showed NECTIN4 positivity in 96% and 99%, with 88% and 83% moderate/strong expressing specimens, respectively, whereas significantly lower NECTIN4 levels were detected in mixed TaHG lesions (72% positivity, 48% of samples with moderate/strong NECTIN4 expression). Moreover, higher NECTIN4 heterogeneity was observed in patients with multifocal mixed TaHG tumors (22% of patients) compared to patients with multifocal CIS/T1HG and pure TaHG/T1HG tumors (9% and 5%). Taken together, NECTIN4-directed antibody–drug conjugates might be promising for the treatment of HR NMIBC patients, especially for those exhibiting CIS/T1HG and pure TaHG/T1HG tumors without a history of low-grade disease.

**Supplementary Information:**

The online version contains supplementary material available at 10.1007/s00428-022-03328-1.

## Introduction

With 549,000 new cases and 200,000 deaths in 2018 worldwide, bladder cancer (BC) is the most common malignancy of the urinary tract [1]. Muscle-invasive bladder cancer (MIBC) has an unfavorable prognosis and develops from high-risk (HR) non-invasive lesions, i.e., from flat-growing carcinoma in situ (CIS) and papillary high-grade (HG) tumors [2]. According to the current treatment guidelines by the European Association of Urology (EAU) and the American Urological Association (AUA) [3, 4], adjuvant intravesical bacillus Calmette-Guérin (BCG)-based immunotherapy following transurethral resection (TUR) of the tumor is the recommended first-line treatment for high-risk (HR) non-MIBC (NMIBC). Unfortunately, BCG therapy fails in a substantial fraction of patients [5] due to resistance or toxicity [6, 7]. It is estimated that approximately 40% of patients treated by BCG will eventually experience a recurrence [7]. Radical cystectomy (RC) is indicated as second-line therapy in case of BCG failure [3, 4]. However, due to its morbidity, not all patients are eligible or refuse RC [7]. Recently, the U.S. Food and Drug Administration (FDA) has approved the immune checkpoint inhibitor (ICI) pembrolizumab for BCG-unresponsive NMIBC, based on results from the KEYNOTE-057 trial (www.clinicaltrials.gov; NCT02625961), improving treatment options for this patient group. However, there is still a high need for alternative bladder-preserving therapies as not all BCG-unresponsive patients respond to immune checkpoint inhibition as evidenced by a complete response rate (CRR) of 41% after more than two-year follow-up from the KEYNOTE-057 trial [8].

NECTIN4 is a type I transmembrane protein that is found overexpressed (in comparison to the respective normal tissue) at the cell surface of several human epithelial malignancies, including cancers of the urinary bladder, breast and lung [9]. It has been demonstrated that the vast majority of advanced urothelial carcinomas overexpress NECTIN4 and that this protein has great potential as a therapeutic target for patients with advanced-stage urothelial carcinoma [10]. In December 2019, the FDA approved the antibody–drug conjugate (ADC) enfortumab vedotin (EV) as a new therapy for patients with locally advanced or metastatic urothelial cancer that have previously been treated with platinum and checkpoint inhibitor therapy. Shortly after, the combination of EV and the ICI pembrolizumab was approved by the FDA as first-line treatment for cisplatin-ineligible patients with locally advanced or metastatic urothelial cancer [11]. EV consists of a human monoclonal NECTIN4-specific antibody attached via a linker to the cytotoxic microtubule-disrupting payload MMAE (monomethyl auristatin E), triggering cell cycle arrest and apoptosis in NECTIN4-positive cells [11].

Far less is known about the expression pattern of NECTIN4 and its potential as a therapeutic target for HR NMIBC. In the current study we aimed to systematically analyze the expression pattern of NECTIN4 in urothelial HR NMIBC by making use of cohorts for CIS, HG papillary tumors (with and without a history of low-grade (LG) disease) as well as stroma-invasive carcinomas. Moreover, we studied the inter-lesional heterogeneity of NECTIN4 expression in patients with multifocal HR NMIBC.

## Materials and methods

### Tissue cohorts

To systematically study NECTIN4 protein expression in subentities of urothelial HR NMIBC, three carefully selected formalin-fixed, paraffin-embedded (FFPE) tumor tissue cohorts were applied, all comprising samples from HR NMIBC patients without a known history of prior or concomitant MIBC. The CIS/T1HG cohort has recently been described in detail [12] and comprised 182 samples from 90 patients diagnosed with CIS, with or without concurrent Ta and T1 HR NMIBC. The number of different bladder tumor locations analyzed per patient ranged from 1 to 7. The second FFPE cohort encompassed 98 samples from 53 patients with HG papillary urothelial BC without a known history of prior or concomitant papillary LG disease (pure TaHG/T1HG) and has been described recently [13]. The number of different bladder tumor locations analyzed per patient ranged from 1 to 6. The third cohort consisted of 87 HG and 95 LG FFPE samples from 82 patients diagnosed with mixed-grade papillary urothelial BC (mixed TaLG/HG). The number of different bladder tumor locations analyzed per patient ranged from 1 to 3. Where available, several tissue cores taken from the same bladder tumor location were analyzed to account for intra-lesional heterogeneity (range: 1–2 (CIS/T1HG), 1–7 (pure TaHG/T1HG), 1–8 (mixed TaLG/HG)). Anonymized patient characteristics for all three tumor tissue microarray (TMA) cohorts are summarized in Table 1. Additionally, NECTIN4 protein levels in normal urothelium were analyzed in an independent cohort comprising 26 samples from 22 patients (Online Resource 1). There was no patient overlap between the independent TMA cohorts. TMAs were composed of 9 (CIS/T1HG), 4 (pure TaHG/T1HG), 12 (mixed TaLG/HG) and 1 (normal urothelium) TMA slide(s). This retrospective immunohistochemical study was performed on FFPE tissue samples from the archives of the Institute of Pathology, RWTH Aachen and the Institute of Pathology, Helios University Hospital Wuppertal and was approved by the Ethics Committee of the University of Witten/Herdecke (No. 55/2019) and the Ethics Committee at the RWTH Aachen Faculty of Medicine (EK 011/21).

**Table 1:** Patient characteristics for the analyzed FFPE tumor tissue cohorts

		Overall	CIS/T1HG cohort	pure TaHG/papillary T1HG	Mixed TaLG/HG
Patients		225	90	53	82
Age (years)					
	median	72	72	76	68
	range	37–93	44–89	37–93	44–92
		n (%)	n (%)	n (%)	n (%)
Gender					
	female	28 (12.4)	12 (13.3)	7 (13.2)	9 (11.0)
	male	198 (87.6)	78 (86.7)	46 (86.8)	73 (89.0)
Tumor stage					
	Ta/CIS	93 (41.2)	45 (50.0)	11 (20.8)	37 (45.1)
	T1	132 (58.4)	45 (50.0)	42 (79.2)	45 (54.9)
Focality					
	unifocal	71 (31.4)	19 (21.1)	19 (35.8)	32 (39.0)
	multifocal	155 (68.6)	71 (78.9)	34 (64.2)	50 (61.0)
Prior treatment					
	BCG	7 (3.1)	6 (6.7)	1 (1.9)	0
	MMC	3 (1.3)	1 (1.1)	2 (3.7)	0
	BCG, MMC	2 (0.9)	0	2 (3.7)	0
	none	133 (58.8)	82 (91.1)	46 (85.2)	5 (6.1)
	unknown	80 (35.4)	1 (1.1)	2 (3.7)	77 (93.9)
Sample source					
	TURBT/biopsy	226 (100.0)	90 (100.0)	53 (100.0)	82 (100.0)
	Cystectomy	0	0	0	0

BCG: Bacillus Calmette-Guérin; MMC: Mitomycin C; TURBT: transurethral resection bladder tumor

### Immunohistochemistry

FFPE material was used to create TMAs, including positive and negative staining controls. TMA sects. (2 µm) were incubated with antigen retrieval solution PT Link (Dako, Agilent) of pH 6 (ERBB2, GATA3, KRT20) or pH 9 (NECTIN4) at 95 °C for deparaffinization, rehydration and epitope retrieval. Slides were subsequently transferred to an automated immunostainer (Dako, Agilent) and covered with EnVision™ Flex Peroxidase Blocking-Reagent (Dako, Agilent) for five minutes. Next, immunostaining was performed using a validated antibody for NECTIN4 (Abcam, ab192033) as well as antibodies for ERBB2, GATA3 and KRT20 that have recently been validated and applied [14]. Immunohistochemical data for ERBB2, GATA3 and KRT20 for the CIS/T1HG and the pure TaHG/T1HG cohort have been available prior to this study and were re-analyzed for the current work [12, 13]. Subsequently, tissue sections were treated with a secondary reagent (Dako, Agilent) for 15 min (except for NECTIN4 staining), followed by incubation with a horseradish peroxidase-conjugated polymer (Dako, Agilent) for 20 min. Finally, visualization of staining was accomplished using a DAB + Substrate Chromogen System (Dako, Agilent), and tissue sections were counterstained using Mayer’s hematoxylin.

All immunohistochemical stainings were assessed by two experienced uropathologists (RK, NTG). The accordance was high (95%) for all proteins analyzed. In case of deviating scores, consensus results have been achieved by choosing unequivocal areas for evaluation. Cytoplasmic and membranous NECTIN4 protein expression was considered and an immunohistochemical score (H-score) was applied, defined as the sum of the products of staining intensity (score of 0–3) and the percentage of stained cells (0–100) at a given intensity [9]. As membranous NECTIN4 positivity is required for delivery of the ADC EV to target cells, membranous NECTIN4 staining was determined separately for each sample. Specimens showing membranous staining of all tumor cells and those exhibiting only partial positivity of a fraction of cancer cells were considered “positive “ for membranous NECTIN4, based on the fact that the EV-associated cytotoxic payload MMAE is membrane-permeable and thus able to diffuse into surrounding antigen-negative cells to trigger bystander-killing [15]. To account for potential intra-lesional heterogeneity per urinary bladder location, several tissue cores of the same tumor material were analyzed (if available) and the mean H-score was calculated. Samples were finally classified as “negative” (H-score 0–14), “weak” (H-score 15–99), “moderate” (H-score 100–199) and “strong” (H-score 200–300). Concerning membranous NECTIN4 staining when analyzing intra-lesional heterogeneity, the tumor sample was overall classified as “membranous-positive” if at least one area of the same tumor material exhibited membranous NECTIN4 positivity. Moreover, inter-lesional, i.e., intra-entity-specific NECTIN4 heterogeneity of multifocal tumors, was considered by analyzing NECTIN4 in independent tumor biopsies taken from different tumor locations in the same urinary bladder, respectively. Assessment of staining results for KRT20, GATA3 and ERBB2 has been reported recently [12]. In brief, for cytoplasmic KRT20, the percentage of positively stained tumor cells was evaluated, whereas nuclear GATA3 was assessed using an adapted semi-quantitative immunoreactive score (IRS) [16]. A semi-quantitative scoring system was applied to quantify ERBB2 expression [17]. To consider intra-lesional heterogeneity, several tissue cores of the same tumor material were analyzed (if available) and the mean staining result was calculated for KRT20 and GATA3, whereas the highest ERBB2 score determined the overall staining result.

### Statistics

To compare protein levels between two groups, the following statistical tests were applied: nonparametric Mann–Whitney test (non-normally distributed, unpaired data), t-test (normally distributed, unpaired data), nonparametric Wilcoxon matched-pairs signed rank test (non-normally distributed, paired data) and a paired t-test (normally distributed, paired data). Comparisons between more than two unpaired groups were accomplished by applying a nonparametric Kruskal–Wallis test and a Dunn’s multiple comparison post-hoc test. To assess Gaussian distribution of data sets, Shapiro–Wilk normality tests were conducted. To analyze potential correlations between NECTIN4 protein and luminal marker expression, the Spearman correlation coefficient was calculated. P values < 0.05 were considered statistically significant. All box plots were generated using GraphPad Prism 9 (v.9.3.1). All analyses were performed by using IBM SPSS Statistics (version 26) and GraphPad Prism 9 (v.9.3.1).

## Results

**Fig. 1:**
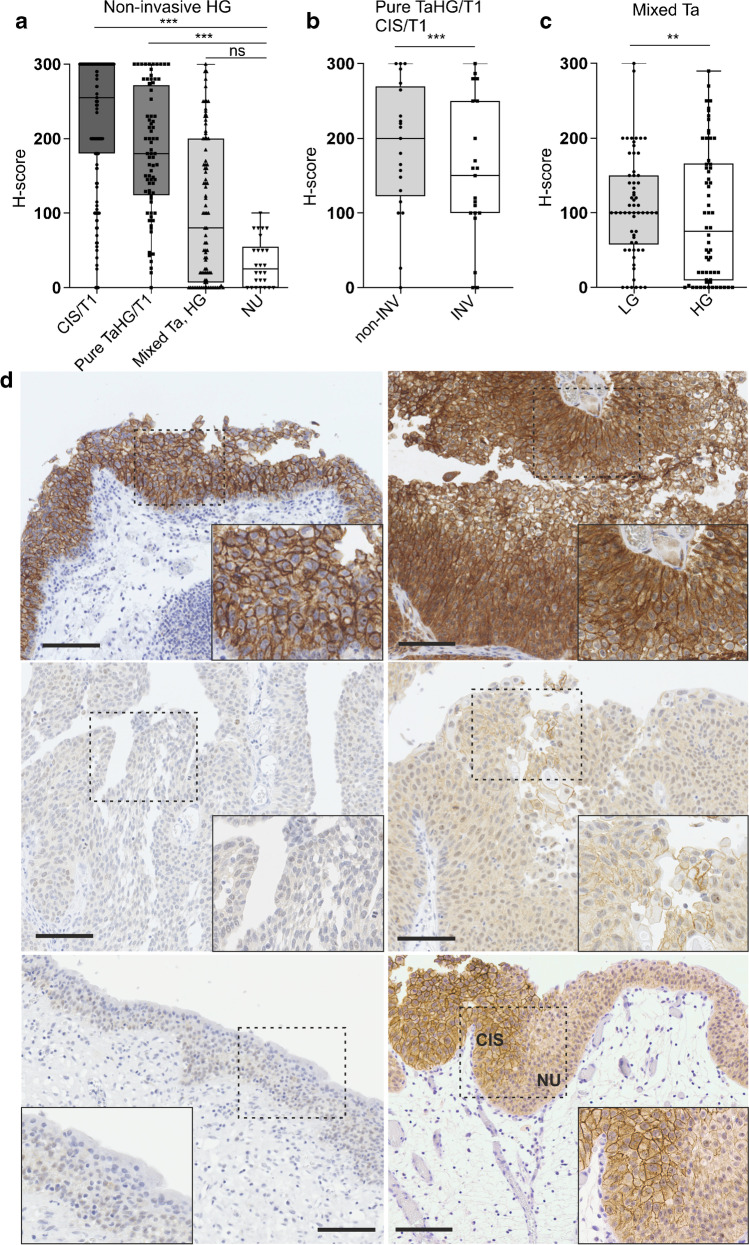
Representative immunohistochemical NECTIN4 staining in HG NMIBC. (**a**) NECTIN4 protein expression in non-invasive areas of samples of different HG NMIBC subentities, i.e., CIS/T1HG (N = 179), pure TaHG/T1HG (N = 76), TaHG from mixed-grade lesions (N = 87) in comparison to expression in normal urothelium (NU) samples (N = 26). ***: P < 0.001, ns = not significant (Kruskal–Wallis test, Dunn’s multiple comparison test). For reasons of clarity, not all significant differences are depicted: all but one (i.e., TaHG from mixed-grade lesions vs. NU samples) comparison are significant at a level of at least P < 0.01. (**b**) Comparison of NECTIN4 protein levels in non-invasive (non-INV) and stroma-invasive (INV) areas (N = 21 matched pairs from the same biopsies) of stroma-invasive pure papillary HG tumors (pure TaHG/T1HG) and CIS/T1HG lesions. ***: P < 0.001 (Paired t-test). (**c**) Comparison of NECTIN4 protein expression in LG and HG regions of mixed-grade papillary tumors (N = 62 matched pairs from the same biopsies). **: P < 0.01 (Wilcoxon matched-pairs signed rank test). (**d**) Representative images showing immunohistochemical NECTIN4 expression in different urothelial HG NMIBC subentities and normal urothelium. Upper panel: strong NECTIN4 expression in non-invasive areas of a CIS/T1HG (left) and a pure TaHG/T1HG sample (right). Middle panel: negative (left) and weak NECTIN4 expression (right) in two TaHG samples from mixed-grade papillary lesions. Lower panel: negative (left) and weak (right) NECTIN4 expression in normal urothelial cells without (left) and with adjacent CIS (right) showing strong NECTIN4 positivity. Boxed areas in each micrograph are shown in higher magnification. Scale bars: 100 μm. CIS: carcinoma in situ, HG = high-grade, INV = invasive, LG = low-grade, NU = normal urothelium

To systematically study the expression pattern of NECTIN4 protein in the heterogenous group of urothelial HR NMIBC, three tissue cohorts for CIS, papillary tumors of mixed grade as well as pure papillary HG tumors, without a history of previous or concomitant LG disease, were analyzed. Additionally, NECTIN4 protein expression in HG NMIBC subentities was compared to NECTIN4 protein levels in normal urothelial samples. NECTIN4 staining results are summarized in Table 2 and Fig. 1.

**Table 2:** Summarized NECTIN4 protein expression in urothelial HG NMIBC samples

		NECTIN4 staining (H-score)					
		Strong N (%)	Moderate N (%)	Weak N (%)	Negative N (%)	Overall positivity^a^ N (%)	Membranous positivity^b^ N (%)
Non-invasive (N = 437)							
	CIS/T1HG, (N = 179)	128 (72)	30 (17)	14 (8)	7 (4)	172 (96)	165 (92)
	Pure TaHG/T1HG (N = 76)	34 (45)	29 (38)	12 (16)	1 (1)	75 (99)	73 (96)
	Mixed Ta, HG (N = 87)	22 (25)	20 (23)	21 (24)	24 (28)	63 (72)	60 (69)
	Mixed Ta, LG (N = 95)	10 (11)	44 (46)	27 (28)	14 (15)	81 (85)	67 (71)
Stroma-invasive (N = 25)							
	CIS/T1HG (N = 3)	1 (33)	2 (67)	0	0	3 (100)	1 (33)
	Pure TaHG/T1HG (N = 22)	6 (27)	10 (45)	4 (18)	2 (9)	20 (91)	15 (68)
NU (N = 26)		0	1 (4)	15 (58)	10 (38)	16 (62)	1 (4)

NECTIN4 protein expression in samples of different HG subentities from HR NMIBC patients and in normal urothelial controls. NECTIN4 expression is classified as „strong “, „moderate “, „weak “ and „negative “, according to the H-score [9]

^a^ comprises all samples showing NECTIN4 expression, except for those classified as „negative “; ^b^ all samples are considered, including those classified as „negative “ for NECTIN4. HG = high-grade, LG = low-grade, NU = normal urothelium

A high overall positivity as well as high membranous positivity was observed for non-invasive areas of urothelial CIS/T1HG (overall positivity: 96%, membranous positivity: 92%, median H-score: 255, interquartile range (IQR) H-score: 300–180) and pure TaHG/T1HG lesions (overall positivity: 99%, membranous positivity: 96%, median H-score: 180, IQR H-score: 272–124) (Table 2 and Fig. 1). In contrast, a significantly lower NECTIN4 protein expression was noted in HG tumors from mixed-grade papillary lesions compared to both aforementioned HG tumor groups (overall positivity: 72%, membranous positivity: 69%, median H-score: 80, IQR H-score: 200–7; for both comparisons: P < 0.001) (Table 2 and Fig. 1).

In comparison to NECTIN4 protein levels in normal urothelial samples (overall positivity: 62%, membranous positivity: 4%, median H-score: 25, IQR H-score: 55–0), NECTIN4 expression in non-invasive parts of CIS/T1HG and of pure TaHG/T1HG lesions was significantly enriched (for both comparisons P < 0.001), but not in HG tumors from mixed-grade papillary lesions (Table 2 and Fig. 1A). Of relevance, a significant reduction in NECTIN4 expression in the stroma-invasive tumor areas was observed when comparing matched pairs of non-invasive (median H-score: 200, IQR H-score: 270–123) and adjacent stroma-invasive areas (median H-score: 150, IQR H-score: 250–100) from the same CIS/T1HG and pure TaHG/T1HG tissue samples (P < 0.001, Fig. 1B). The difference in NECTIN4 protein expression between normal urothelial and stroma-invasive areas from CIS/T1HG and pure TaHG/T1HG samples remained significant (P < 0.001, data not shown). Moreover, increased median NECTIN4 protein levels were noted in LG tumor areas when comparing LG to matched HG tumor samples of mixed-grade lesions (P < 0.01, Fig. 1C).

**Fig. 2:**
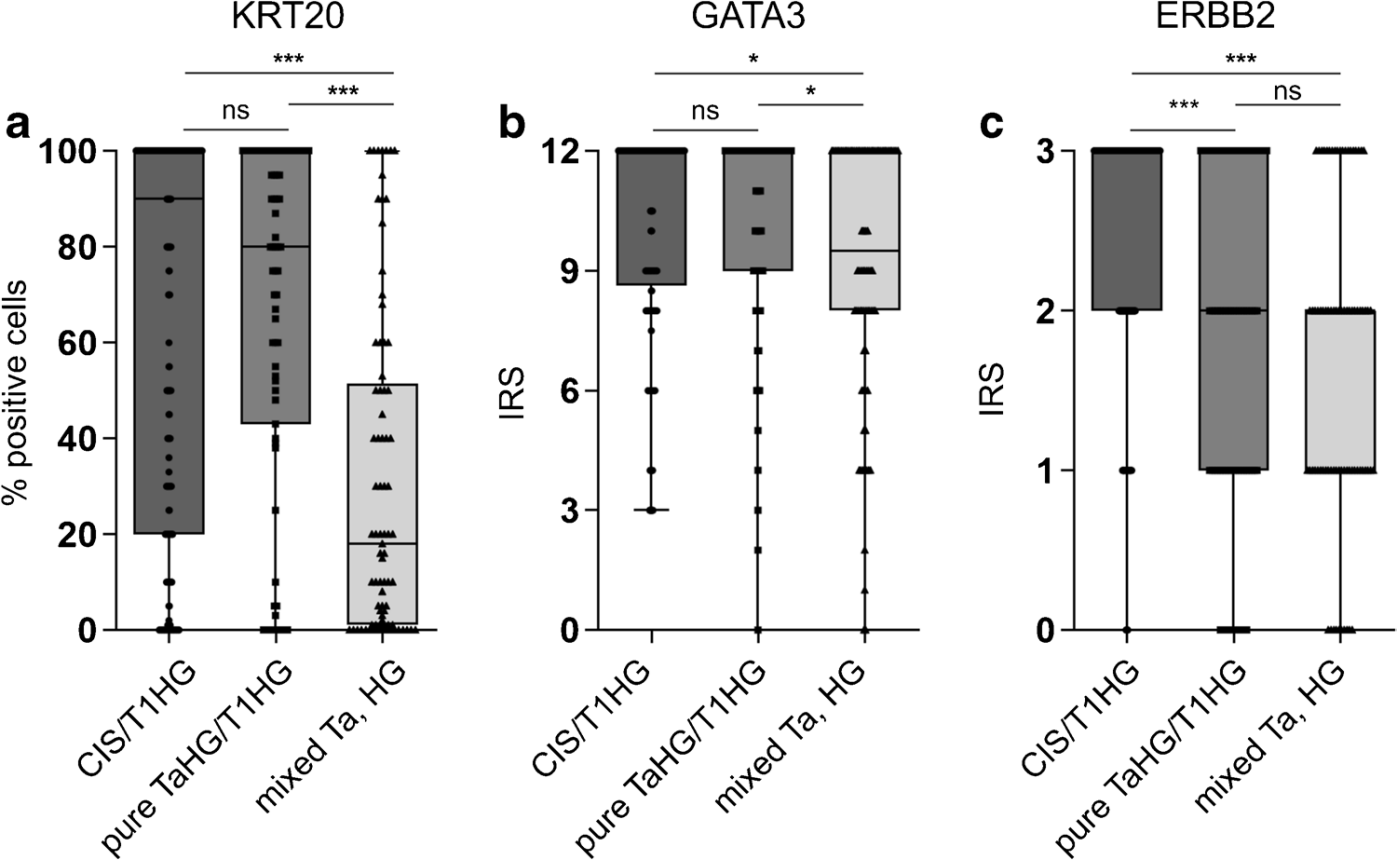
Luminal marker expression in urothelial NMIBC HG subentities. Depicted are the protein levels for the luminal markers (**a**) KRT20, (**b**) GATA3 and (**c**) ERBB2 in non-invasive areas of CIS/T1HG, pure TaHG/T1HG and papillary HG lesions from mixed-grade tumors (mixed Ta, HG). The analyzed sample numbers are: KRT20 (CIS/T1HG: 204, pure TaHG/T1HG: 75, mixed Ta, HG: 81), GATA3 (CIS/T1HG: 204, pure TaHG/T1HG: 73, mixed Ta, HG: 80), ERBB2 (CIS/T1HG: 206, pure TaHG/T1HG: 75, mixed TaHG: 79). ***: P < 0.001, *: P < 0.05, ns = not significant (Kruskal–Wallis test, Dunn’s multiple comparison test). IRS = immunoreactive score

Recently, it has been reported that NECTIN4 expression is significantly enriched in luminal subtypes of MIBC [18]. To analyze this observation in NMIBC, the expression of luminal markers (KRT20, GATA3 and ERBB2) in the mentioned NMIBC HG subentities was compared (Fig. 2). The lowest expression of luminal markers was found in the group of papillary HG tumors from mixed-grade lesions (exhibiting the lowest NECTIN4 protein levels). Compared to this group, protein levels of KRT20 and GATA3 were significantly enriched in non-invasive areas of CIS/T1HG and of pure TaHG/T1HG lesions (for KRT20: P < 0.001, for GATA3: P < 0.05) (Fig. 2). Correlating NECTIN4 protein amounts with luminal marker expression across NMIBC HG subgroups, a weak positive correlation was noted between NECTIN4 and marker protein levels (for all: two-sided P < 0.01; Spearman r (ERBB2: 0.323; KRT20: 0.234; GATA3: 0.193)) (Online Resource 2).

Taken together, NECTIN4 protein expression was significantly enriched in luminal-like urothelial CIS/T1HG and pure papillary TaHG/T1HG tumor samples compared to normal urothelial NECTIN4 protein levels, while this was not true for papillary HG lesions from mixed-grade tumors, showing markedly lower NECTIN4 and luminal marker protein levels.

Consideration of intra-patient heterogeneity of therapeutic target expression is of high importance with regard to therapy response, as recently shown for ERBB2 [19, 20]. Therefore, to study intra-entity heterogeneity of NECTIN4 protein expression, independent biopsies of the same tumor entity, taken from distinct locations in the urinary bladder of HR NMIBC patients with multifocal disease, were analyzed (Table 3).

**Table 3:** NECTIN4 protein expression in multifocal urothelial HR NMIBC patients

	Overall staining pattern			Membranous staining		
Entity (Patients)	Positive N (%)	Negative N (%)	Heterog N (%)	Positive N (%)	Negative N (%)	Heterog N (%)
CIS/T1HG (N = 47)	43 (91.5)	0	4 (8.5)	42 (89.4)	4 (8.5)	1 (2.1)
Pure TaHG/T1HG (N = 22)	21 (95.5)	0	1 (4.5)	19 (86.4)	1 (4.5)	2 (9.1)
Mixed TaLG/HG (N = 18)	12 (66.7)	2 (11.1)	4 (22.2)	9 (50.0)	3 (16.7)	6 (33.3)

Intra-entity-specific heterogeneity of NECTIN4 protein expression in different HG NMIBC subentities was considered by analyzing independent tumor biopsies of the same HG subentity (range: 2–7 (CIS), 2–6 (pure TaHG/T1HG), 2–3 (mixed TaLG/HG)) taken from different locations in the same urinary bladder, respectively. For multifocal mixed TaLG/HG tumors, only staining of the HG portion was considered. Heterog: independent biopsies of the same patient show heterogenous (i.e., negative and positive) NECTIN4 protein expression (based on H-score [9])

A higher overall NECTIN4 positivity (92% and 96%) and membranous positivity (89% and 86%) was noted in HR NMIBC patients with multifocal CIS/T1HG and in patients with multifocal pure TaHG/T1HG lesions compared to patients exhibiting multifocal papillary HG tumors associated with LG lesions (overall NECTIN4 positivity: 67%, membranous positivity: 50%) (Table 3). Moreover, a markedly higher degree of heterogeneity in NECTIN4 protein expression was observed in multifocal TaHG tumors from mixed-grade lesions (heterogenous overall NECTIN4 staining: 22%, heterogenous membranous NECTIN4 staining: 33%) in comparison to multifocal CIS/T1HG (heterogenous overall staining: 9%, heterogenous membranous staining: 2%) and pure TaHG carcinomas (heterogenous overall staining: 5%, heterogenous membranous staining: 9%) (Table 3).

## Discussion

It has been revealed that the cell surface protein NECTIN4 is a cancer-associated antigen, with the majority of human bladder carcinomas expressing moderate to high NECTIN4 protein levels (60%), while normal urothelial tissues exhibit lower (weak to moderate) NECTIN4 protein amounts [9]. Recently, the NECTIN4-directed ADC EV has been approved by the FDA for the treatment of locally advanced and metastatic urothelial cancer [11]. However, far less is known about the expression pattern of NECTIN4 in early stages of urothelial BC. In this study, we systematically addressed this aspect by focusing especially on the heterogenous group of urothelial HR NMIBC, representing a subgroup of patients with only limited treatment options [3].

In the current study, we observed a high prevalence of NECTIN4 protein positivity in non-invasive areas of urothelial HG bladder tumor samples, including CIS and papillary HG tumors with and without a history of LG disease (91%, N = 342), with a relatively high level of NECTIN4 protein expression (77% of samples showing moderate to strong NECTIN4 protein levels according to the recently described H-score groups [9]). Similar observations have been made for stroma-invasive parts of CIS/T1HG and pure TaHG/T1HG lesions (N = 25), with a portion of 92% NECTIN4 overall protein positivity and moderate to high NECTIN4 protein expression levels in 76% of samples.

In a recently published work, comprising a small cohort of urothelial NMIBC samples (N = 83) and that was not specifically focused on HG urothelial BC, a slightly lower overall NECTIN4 protein positivity of 87% was noted [21]. Moreover, in the current study, a so far unreported heterogenous NECTIN4 protein expression pattern was noted between subgroups of urothelial HG bladder tumors that might be relevant with regard to therapy response of HR NMIBC patients: While non-invasive areas of CIS/T1HG and pure TaHG/T1HG tumors showed an overall NECTIN4 positivity in 96% and 99% of samples, with 88% and 83% moderate to strong expressing specimens, respectively, significantly lower NECTIN4 levels were detected in papillary HG tumors from mixed-grade lesions (72% overall positivity, 48% of samples with moderate to strong NECTIN4 expression). Additionally, we observed a significant reduction in NECTIN4 protein expression in stroma-invasive tumor areas compared to matched non-invasive areas of the same CIS/T1HG and pure TaHG/T1HG tissue samples, respectively. This is in accordance with recently observed lower *NECTIN4* mRNA levels in pT1 compared to pT2 tumors [18] and should be further analyzed as potentially relevant regarding enfortumab vedotin response.

Despite a reported high prevalence of NECTIN4 overall protein positivity (of nearly 100% of patient biopsies) in clinical cohorts, comparatively low response rates (43% and 44% objective response rates) have been observed in clinical EV trials [10, 22], suggesting that additional predictive markers and/or adjustment of NECTIN4 assessment is needed to more accurately predict therapy response. In this regard, a recent BC study has noted that *NECTIN4* expression is positively correlated with luminal marker expression and enriched in luminal BC subtypes [18]. In agreement with this observation, we have noted a weak but significant positive correlation between NECTIN4 and luminal marker (KRT20, GATA3 and ERBB2) expression across HG NMIBC subentities, with lowest NECTIN4 and luminal marker amounts in papillary HG tumors from mixed-grade lesions.

In addition to analyzing the overall NECTIN4 positivity (i.e., cytoplasmic and/or cell membranous staining), cell membrane-localized NECTIN4, the target structure for EV binding [9], was separately scored for all samples. Even though no large differences between overall NECTIN4 staining and NECTIN4 cell membrane positivity have been observed in non-invasive areas of urothelial HG BC lesions, a prominent discrepancy was present in the stroma-invasive parts of urothelial HG BC samples (96% overall NECTIN4 protein positivity, 51% membranous NECTIN4 positivity) as well as normal urothelial specimens (62% overall protein positivity, 4% membranous NECTIN4 positivity). If membranous positivity might be associated with therapy response of BC patients, needs to be addressed in future clinical trials.

Moreover, intra-entity-specific heterogeneity of NECTIN4 protein expression was considered by analyzing independent biopsies of the same tumor entity, taken from distinct locations in the urinary bladder of HR NMIBC patients with multifocal disease. Of note, while NECTIN4 inter-lesional heterogeneity was relatively low in patients with multifocal CIS/T1HG and patients with multifocal pure TaHG/T1HG tumors, a higher degree of NECTIN4 heterogeneity was observed in patients with multifocal TaHG tumors from mixed-grade lesions. It has been reported for ERBB2, another therapeutic target of ADC-based therapies, that higher intra-tumoral heterogeneity found by *ERBB2*-FISH in ERBB2-positive breast cancer patients treated by an anti-ERBB2 therapy, correlated with worse outcome compared to those without ERBB2 heterogeneity [20].

This study focused on HR NMIBC patients for which BCG therapy is the only recommended intravesical therapy by the European Association of Urology (EAU) at the moment [3]. Results from a currently recruiting (NCT05014139, https://clinicaltrials.gov/) and additional upcoming enfortumab vedotin trials in the HR NMIBC setting will hopefully soon shed light on effectiveness and side effects of enfortumab vedotin treatment in this disease entity.

The current study has several limitations: the normal urothelium cohort mainly comprises normal urothelium samples from patients without bladder carcinoma and thus without adjacent HG bladder cancer. As data on NECTIN4 expression in normal urothelium are scarce, further studies should investigate NECTIN4 levels in normal urothelium of bladder cancer patients as possibly relevant regarding potential side effects of enfortumab vedotin treatment. Moreover, data on prior therapy that might influence NECTIN4 levels are not available for all cases in our cohorts. However, recent data indicate that BCG therapy might not significantly impact protein levels of NECTIN4 [23]. Lastly, immunohistochemical stainings have been quantified by two experienced uropathologists. However, unlike for automated image analysis, a cognitive bias in the quantified data can thus not fully be excluded.

Taken together, these preliminary data, in addition to those from previous reports [13, 14], suggest that NECTIN4, as well as ERBB2-directed ADC-based therapies, might be promising for the treatment of HR NMIBC patients, especially for those patients exhibiting luminal-like CIS/T1HG and pure papillary HG tumors without a history of LG disease.

## Supplementary Information

Below is the link to the electronic supplementary material.

Supplementary file1 (XLSX 12 KB)

Supplementary file2 (PDF 42 KB)

## Data Availability

All data underlying the reported findings are included within the manuscript and its supplements. Anonymized raw datasets generated during the current study are available from the corresponding author on reasonable request.
