# Supplementary material for: Heterogenous NECTIN4 expression in urothelial high-risk non-muscle-invasive bladder cancer
Source: Virchows Arch. 2022 Apr 28;481(1):83–92. doi: 10.1007/s00428-022-03328-1 (PMC9226103; doi:10.1007/s00428-022-03328-1)
Supplement: Supplementary file 2 — Supplementary file2 (PDF 42 KB) [file 428_2022_3328_MOESM2_ESM.pdf]

Virchows Archiv

Heterogenous NECTIN4 expression in urothelial high-risk non-muscle -invasive bladder cancer

Stefan Garczyk, Stephan Degener, Felix Bischoff, Tician Schnitzler, Anne Salz, Reinhard Golz, Alexander Buchner, Gerald B. Schulz, Ursula Schneider, Nadine T. Gaisa, Ruth Knüchel

Corresponding author:

Stefan Garczyk, PhD, Institute of Pathology, University Hospital RWTH Aachen, Pauwelsstr. 30, 52074 Aachen, Germany,

Email: sgarczyk@ukaachen.de, ORCID: 0000-0001-5447-3207

Online Resource 2 Correlation of NECTIN4 protein amounts with luminal marker expression across all NMIBC HG subgroups

			NECTIN4	KRT20
Spearman-Rho	NECTIN4	Correlation coefficient	1.000	.234**
		Sig. (2-sided)	.	.000
		N	342	321
	KRT20	Correlation coefficient	.234**	1.000
		Sig. (2-sided)	.000	.
		N	321	360

			NECTIN4	ERBB2
Spearman-Rho	NECTIN4	Correlation coefficient	1.000	.323**
		Sig. (2-sided)	.	.000
		N	342	326
	ERBB2	Correlation coefficient	.323**	1.000
		Sig. (2-sided)	.000	.
		N	326	360

			NECTIN4	GATA3
Spearman-Rho	NECTIN4	Correlation coefficient	1.000	.193**
		Sig. (2-sided)	.	.001
		N	342	325
	GATA3	Correlation coefficient	.193**	1.000
		Sig. (2-sided)	.001	.
		N	325	357

**: P < 0.01 (two-sided)
